# Intention Seekers: Conspiracist Ideation and Biased Attributions of Intentionality

**DOI:** 10.1371/journal.pone.0124125

**Published:** 2015-05-13

**Authors:** Robert Brotherton, Christopher C. French

**Affiliations:** Department of Psychology, Goldsmiths, University of London, London, United Kingdom; Universitat Wien, AUSTRIA

## Abstract

Conspiracist beliefs are widespread and potentially hazardous. A growing body of research suggests that cognitive biases may play a role in endorsement of conspiracy theories. The current research examines the novel hypothesis that individuals who are biased towards inferring intentional explanations for ambiguous actions are more likely to endorse conspiracy theories, which portray events as the exclusive product of intentional agency. Study 1 replicated a previously observed relationship between conspiracist ideation and individual differences in anthropomorphisation. Studies 2 and 3 report a relationship between conspiracism and inferences of intentionality for imagined ambiguous events. Additionally, Study 3 again found conspiracist ideation to be predicted by individual differences in anthropomorphism. Contrary to expectations, however, the relationship was not mediated by the intentionality bias. The findings are discussed in terms of a domain-general intentionality bias making conspiracy theories appear particularly plausible. Alternative explanations are suggested for the association between conspiracism and anthropomorphism.

## Introduction

Conspiracy theories, while not false by definition, are characteristically unverified, implausible, and epistemically unsound [1]. Despite this, belief in conspiracy theories is widespread [2–7]. Moreover, conspiracy theories can have tangible consequences for believers, and for the wider community. Conspiracy theories about HIV/AIDS and vaccines can influence health-related behaviour [8–11], theories about anthropogenic climate change can influence attitudes towards the environment [12], and theories about governmental malfeasance can foster radicalisation and violence [13].

Until recently, conspiracism has been largely neglected by psychologists. However, a literature is now beginning to emerge pointing towards individual differences and cognitive factors which may be associated with endorsement of conspiracy theories, including such variables as agreeableness, authoritarianism, openness, mild paranoia, confirmation bias, the conjunction fallacy, illusory pattern perception, the proportionality bias, and projection [3,5,14–24]. The current research concerns a psychological factor which has yet to be explored in depth: attributions of intentionality. Conspiracy theories offer to explain complex and often ambiguous events in terms of intentional agency. This research is guided by the hypothesis that, to the extent that an individual tends to regard ambiguous events or situations generally as having been intended, conspiracy theories may appear more plausible than alternative explanations.

### (Over)attributing intent

Everyday social interaction depends on judgements of intentionality. This refers to the ability to distinguish intentional actions and consequences from unintentional acts or outcomes, and to infer the specific intentions motivating people’s actions. Judgements of intentionality are integral to understanding and participating in routine social interactions such as conversation [25] and interpreting a particular individual’s behaviour over time [26,27], as well as understanding more abstract social enterprises such as theatre or literature [28,29]. Deficits in the ability to comprehend the mental states of others can lead to difficulties in everyday life [26].

Given the importance of inferring the intentions of others, it is not surprising that the cognitive system is keenly attuned to intentionality cues. The ability to perceive and infer intentionality appears to be driven by low-level, automatic processes which preferentially encode intent-relevant details while disregarding intent-irrelevant actions [26,30–32]. The cognitive architecture underlying the ability to discern intentionality begins development in early infancy and appears to mature rapidly during early childhood [27,33,34]. By adulthood, most neurotypical individuals share a common understanding of the concept of intentionality, even in lieu of an explicit definition [35,36].

The fast and automatic operation of intentionality-seeking cognitive processes allows us to quickly make inferences about the mental states of those around us—an important evolutionary adaptation [37]. However, as is the case with other low-level cognitive processes [38,39], inferences of intentionality may be subject to biases and heuristics. Not only are we sensitive to the intentions of others, but we may be *overly* sensitive, biased towards perceiving or inferring intentionality even where such an attribution may not be warranted.

An adult who observes another person sneeze, for example, may be *explicitly* aware that the action was unintended. However, research suggests that this awareness is only arrived at secondarily, through effortful application of the acquired knowledge that intentions are not the only possible causes of actions [31,40–42]. In particular, Rosset [31] reports a series of studies suggesting that the low-level processes governing attributions of intentionality initially interpret *all* actions as intentional—even actions which are never performed intentionally, such as catching a cold. Only after the initial automatic attribution of intentionality has been made can higher-level cognitive processes evaluate and, if necessary, override this involuntary assumption. Thus, Rosset found that judging an action to be unintentional requires more cognitive resources, takes longer, and results in increased ease of recall compared to judging the same action to be intentional. Rosset refers to this irresistible inclination towards intentional attributions as the *intentionality bias*.

It must be noted that one attempted replication of Rosset’s studies failed to produce the same results [43]. However, the notion of an intrinsic intentionality bias is consistent with the wider body of research suggesting that intentional explanations are often preferred over unintentional or situational explanations, even when a more tenable unintentional explanation is available. This is especially notable in children under the age of five, but the tendency persists into adulthood [41,42,44–51]. Moreover, adults often readily attribute anthropomorphic intentions to nonhuman animals, or inanimate objects and entities [41,52,53]. Even abstract two-dimensional shapes moving around a screen are often imbued with human-like characteristics and intentions when they move in ways consistent with our expectations of intentional agency [54–56]. Attributions of intentional agency become particularly likely when processing is rushed or disrupted by consumption of alcohol [40,41].

### Intentionality and conspiracy theories

According to conspiracy theories, nothing happens by accident. Such theories invariably explain events in terms of intentional agency, portraying the postulated conspirators as preternaturally competent in their ability to plan and control events, and discounting the role of chance or unintended consequences [1]. Research has yet to examine whether there are measurable individual differences in susceptibility to the intentionality bias—that is, whether some people are habitually less able or inclined to override automatic attributions of intentionality. Yet it seems reasonable to suggest that if some individuals are more susceptible to the intentionality bias, they will tend to find conspiracy theories more plausible than their corresponding mainstream explanations, which are generally more contingent on accidents and unintended consequences.

Lending additional plausibility to the notion that biased attributions of intentionality may play a role in the adoption of conspiracist beliefs, research suggests that other anomalous beliefs are more prevalent among people biased towards unwarranted inferences of intentionality [37,52,57–60]. It is worth noting that belief in conspiracy theories correlates with anomalous beliefs, such as belief in the paranormal, superstition, and New Age beliefs [24,61,62]. Moreover, the tendency to endorse statements postulating some form of supernatural intentionality increases when participants are made to feel powerless [63–65]. Endorsement of conspiracy theories has also been found to increase under conditions of diminished self-efficacy [19,66,67].

To date, few studies have touched upon the speculation that conspiracist beliefs may be a product, in part, of a bias towards attributing intentionality. Imhoff and Bruder [68] report that people who indicated stronger beliefs in conspiracies in general were more likely to blame a specific real-world disaster (the 2011 Fukushima Daiichi nuclear power plant catastrophe) on intentional misconduct, as opposed to chance. However, given the apparent monological nature of conspiracist ideation [3], this correlation may simply represent a proclivity towards assuming ambiguous real-world events were caused by conspiracy, rather than being a product of a more domain-general intentionality bias. Additionally, conspiracist ideation has been found to correlate positively with individual differences in anthropomorphism [68,69].

In sum, there is good reason to suspect a link between conspiracist ideation and promiscuous attributions of intentionality, although such a relationship remains to be demonstrated. Moreover, it is possible that the observed relationship between conspiracism and anthropomorphism may be a result of promiscuous attributions of intent. The current studies examine the hypothesis that individuals biased towards favouring intentional explanations for ambiguous actions in general may see conspiratorial explanations, which paint events as the product of powerful hidden agents’ intentions, as being more plausible than non-conspiracist explanations.

## Study 1: Individual Differences in Anthropomorphisation

Overattribution of intentionality is not limited to ambiguous actions performed by humans. Nonhuman animals or inanimate objects and entities are often attributed human-like intentionality [54–56,70,71]. This tendency is referred to as anthropomorphisation. Individuals differ in their proclivity to anthropomorphise, and these individual differences have been found to influence more specific beliefs, including attributions of responsibility and blame, and feelings of care and concern toward anthropomorphised entities [53]. Some findings indicate that religious beliefs are associated with anthropomorphism [59], suggesting that anomalous beliefs about supernatural agents may appear especially plausible to those who generally see the world as suffused with intentionality. Likewise, two studies have found evidence of a modest but reliable positive association between individual differences in anthropomorphism and conspiracist ideation [68,69]. The current study aimed to replicate the relationship between conspiracist ideation and anthropomorphism using a different measure of generic conspiracism.

### Method

#### Ethics statement

The study was approved by the Goldsmiths, University of London, Department of Psychology Ethics Committee. All participants provided written, informed consent.

#### Participants

Eighty-four undergraduate psychology students at two London-based universities completed the study. No reward was offered for taking part.

#### Design

A correlational design was employed. The variables of interest were individual differences in conspiracist ideation and anthropomorphism.

#### Materials

*Generic conspiracist beliefs* *(GCB)* [72]. Several existing scales measure beliefs in specific conspiracy theories (concerning, for example, the 9/11 attacks and the assassination of John F. Kennedy). However, findings suggest that such beliefs are a product of more abstract assumptions about how the world works (e.g. that governments routinely perpetrate covert acts of terrorism against their own people) [72–74]. Measuring these 'generic' conspriacist beliefs has been shown to provide a valid assessment of individual differences in conspiracist ideation [72]; thus, the GCB was selected as an appropriate scale for the present studies. The scale consists of 15 items (e.g. “New and advanced technology which would harm current industry is being suppressed”) rated on a 5-point scale (1: definitely not true; 2: probably not true; 3: not sure / cannot decide; 4: probably true; 5: definitely true). A single overall conspiracism score was calculated for each participant by averaging their responses to the 15 items. Cronbach’s alpha for the scale was high (.90).

*Individual Differences in Anthropomorphism Questionnaire (IDAQ)* [75]. The IDAQ consists of 15 items assessing the degree to which individuals tend to anthropomorphise nonhuman animals and inanimate objects (example item: “To what extent does a television set experience emotions?”). Participants respond on a Likert-type scale ranging from 1 (“Not at all”) to 10 (“Very much”). Internal reliability in the current study was high (.88).

#### Procedure

Students were approached to take part in research following lectures on unrelated topics. Volunteers were given printed questionnaire packs. The IDAQ was always presented before the GCB. The word ‘conspiracy’ was not mentioned in the information sheet presented to participants prior to filling in the questionnaire. No time limit was given, though participants were asked to work quickly, answering with their first instincts.

### Results

#### Data screening and descriptives

Raw data are available S1 Dataset. No cases were missing data for more than one item. Mean GCB and IDAQ scores were calculated for each participant. One bivariate outlier was excluded from analyses (total valid *N* = 83).

GCB scores were approximately normally distributed. On the whole, participants demonstrated modest scepticism towards conspiracist ideas (*M* = 2.43; *SD* = 0.76). IDAQ scores showed slight positive skew (skewness = 0.70). Participants were generally somewhat disinclined to anthropomorphise, evident from a grand mean somewhat below the mid-point of the scale (*M* = 3.33; *SD* = 1.39).

#### Association between conspiracist ideation and anthropomorphisation

The correlation between mean GCB scores and mean IDAQ scores was moderate and positive (*r* (81) = .39, *p* <. 001). That is, people who endorsed generic conspiracist ideas more strongly tended also to endorse anthropomorphic statements more strongly.

### Discussion

The aim of the present study was to replicate previous research indicating a relationship between individual differences in anthropomorphisation and conspiracist ideation [68,69], using an alternative measure of conspiracism. As expected, a modestly sized positive correlation was found. The findings appear consistent with the idea that conspiracist ideation is associated with promiscuous inferences of intentionality. However, it is unclear whether anthropomorphism reflects a general bias towards inferences of intentionality. Thus, Studies 2 and 3 examine the inter-relationships between conspiracism, anthropomorphism, and the intentionality bias in more detail.

## Study 2: Conspiracist Ideation and Inferences of Intentionality

Having observed a correlation between conspiracism and anthropomorphism, this next study aimed to establish whether conspiracist ideation is related to a domain-general intentionality bias. Previous research suggests that people high in conspiracist ideation are more likely to explain a real-world event as the result of intentional conspiratorial misconduct [68]. The current study, however, presents the first examination of whether people high in conspiracist ideation tend to prefer intentional attributions for ambiguous scenarios *in general*—that is, beyond the context of conspiracy.

### Method

#### Ethics statement

The study was approved by the Goldsmiths, University of London, Department of Psychology Ethics Committee. All participants provided written, informed consent.

#### Participants

A sample of 102 first-year psychology undergraduate students (81 females and 21 males) completed the questionnaire. The majority of participants were of British (62.7%) or other European nationalities (27.5%). Participant age ranged from 18 to 44 years (*M* = 21.0, *SD* = 5.2).

#### Design

A correlational design was employed. The variables of interest were individual differences in conspiracist ideation, and inferences of intentionality.

#### Materials

*Generic conspiracist beliefs (GCB)*. General conspiracist ideation was again assessed using the GCB. Cronbach’s alpha for the scale was high once again (.88).

*Inferences of intentionality*. To measure individual differences in bias towards inferences of intentionality, a measure was adapted from previous research looking at the intentionality bias. Rosset [31] created a list of 34 sentences, each describing an action that can be done either on purpose or by accident. Through pretesting, Rosset ranked the statements in terms of the proportion of participants who offered an intentional explanation for each. Some statements were almost never interpreted as intentional, such as “She burnt the meal.” Some were almost always given intentional explanations, such as “She averted her eyes.” Crucially, however, 12 sentences were more ambiguous, with between 27% and 69% of participants offering intentional interpretations. These 12 sentences were selected as test items for the current study (e.g. “He set the house on fire”; “She kicked the dog”).

Following Rosset’s (Study 2) methodology, two example sentences were presented to familiarise participants with the task requirements. Subsequently, each test sentence was presented together with a space in which participants were asked to write “a brief description of the image that comes to mind when reading each sentence”. On turning the page after writing descriptions for all 12 sentences, participants received additional instructions asking them to “Go back to each of your responses and clarify whether the event you described was done on purpose or by accident.” Participants were required to write the words ‘on purpose’ or ‘by accident’ after each of their descriptions on the previous page. This step was included in the hope of avoiding subjectivity in the coding of descriptions in which the intentions of the actor may be unclear. Rosset gives the example of the following response to the sentence “He dripped paint on the canvas”: “I see a guy in overalls holding a paint brush and looking down at a large canvas on the floor in a loft like building.” Here it is unclear whether the actor intended to drip the paint or not. As per Rosset, participants were not asked for this clarification until after they had completed the open-ended description phase of the task so as to avoid priming participants to think of unintentional explanations which may not have otherwise come to mind.

#### Procedure

Undergraduate students were asked to take part in this research following a lecture on an unrelated topic in return for course credit. The word ‘conspiracy’ was not mentioned in the information sheet given to participants prior to filling in the questionnaire. Two versions of the questionnaire pack, with the order of intentionality items counterbalanced, were randomly distributed. The intentionality measure was always presented before the GCB. No time limit was given, though participants were asked to work quickly, answering with their first instincts.

### Results

#### Data screening

Raw data are available in S1 Dataset. Despite asking participants to clarify whether their descriptions of the ambiguous actions were ‘on purpose’ or ‘by accident’ in the hope of avoiding experimenter subjectivity, examination revealed some seemingly incongruous responses. In some cases a participant’s description of an item suggested an action which was unambiguously accidental, yet they indicated that the action was purposeful, or vice versa. For example, one participant responded to the item “He set the house on fire” with the following description: “I see a man standing outside a burning building holding a petrol can and laughing maniacally.” This would appear to imply an incontrovertibly deliberate act of arson; however, the participant rated the action as accidental.

Out of 1,224 (102 participants multiplied by 12 items each) items total, the lead author identified 30 contentious items (2.45%) such as these across 10 participants. These 30 items, as well as 15 randomly chosen uncontentious control items, were presented to an independent rater who blindly coded each item as contentious or uncontentious. Of the 15 control items which the first rater judged to be uncontentious, the second rater agreed for 13 items (86.7% agreement). Of the 30 items judged by rater 1 to be contentious, rater 2 judged 24 to be contentious (80% agreement). An overall inter-rater reliability analysis using the Kappa statistic was performed to determine consistency between raters, revealing acceptable agreement (Kappa = 0.63; *p* <. 001). Accordingly, the 24 items which both raters judged to be contentious were excluded from subsequent analyses. In addition to excluding individual ambiguous items, a participant’s data were excluded entirely if more than one item was missing (*n* = 5) or contentious (*n* = 7). Four additional multivariate outliers were excluded from analyses (total valid *N* = 86).

#### Descriptive data

Individual intentionality scores were computed by summing the number of intentional explanations each participant offered across the 12 items. Where a participant had a missing or excluded item, their total was adjusted by dividing by 11 and then multiplying by 12 to give a score equivalent to participants with complete data for all 12 items. On the whole, participants tended to offer intentional attributions for significantly more than half (*M* = 7.47; *SD* = 1.52) of the 12 items (*t* (85) = 9.14, *p* <. 001, *d* = 0.99). The data were approximately normally distributed about the mean, with slight negative skew; scores ranged from 4 to 10 (median = 7; skew = -.14).

In addition, a mean conspiracist ideation score was computed for each participant by averaging their responses to the 15 GCB items. On the whole, participants demonstrated modest conspiracist ideation; the overall mean score was 2.90 (*SD* = 0.64), close to the mid-point of the scale. GCB scores were approximately normally distributed about the mean (range = 1.47 to 4.27; median = 2.97; skew = -.14).

#### Association between conspiracist ideation and intentional inferences

There was a small but statistically significant positive correlation between GCB scores and the number of intentional inferences participants offered (*r* (84) = .22, *p* <. 05); that is, participants who endorsed generic conspiracist claims more strongly tended to offer slightly more intentional interpretations.

### Discussion

This study aimed to provide an initial test of the hypothesis that individuals who are more inclined to inferences of intentionality *in general* will view conspiracy theories more favourably. The data were consistent with this hypothesis.

The study raises some issues concerning the operationalisation of individual differences in intentionality inferences that future research using the current measure might take into consideration. In particular, cases of apparent incongruence between the description a participant offered for an item and their explicit rating of ‘on purpose’ or ‘by accident’ suggests that a small minority of participants may not have interpreted the task instructions in the same way as other participants. Ambiguous cases such as these may reflect differing interpretations of the concept of intent. For instance, a pyromaniac may be deemed to not be legally responsible for their actions by virtue of temporary insanity. As far as the current research is concerned, though, the act was intentional: the actor intended to bring about the observed consequences through their actions. Alternatively, some ambiguous responses may simply reflect misunderstanding of the task. Of potential relevance is the fact that 8 of the 9 participants with ambiguous responses indicated non-British nationality or non-Caucasian ethnicity. It is possible that cultural or linguistic nuances affected some participants’ performance on the task. Another possibility is that the apparent incongruence reflects misunderstanding on the part of the rater, rather than the respondent; however, an independent rater was employed to minimise this possibility. Study 3 employed some slight procedural modifications with these issues in mind.

## Study 3: Associations between Anthropomorphisation and Intentional Inferences

Study 1 found a relationship between conspiracism and individual differences in anthropomorphism, and Study 2 found a relationship between conspiracism and individual differences in intentionality bias. It seems plausible to suggest that anthropomorphism itself may be a product of overattribution of intentionality. If that is the case, then anthropomorphism and biased intentionality inferences should be positively related, and the association between anthropomorphism and conspiracism ought to be mediated by biased inferences of intentionality. This final study examined all three variables in conjunction, seeking to clarify the inter-relationships.

### Method

#### Ethics statement

The study was approved by the Goldsmiths, University of London, Department of Psychology Ethics Committee. All participants provided written, informed consent. (n.b. Ethical approval was granted for the collection of data from participants aged 16 years and older in Study 3. All participants under the age of 18 were school children visiting Goldsmiths as part of a field trip, whose guardians provided the school with written consent to take part in the trip; this was not collected by the researchers. Caretakers for the visit provided verbal consent at the time of testing, and participants provided their written, informed consent. This consent procedure was approved by the Goldsmiths, University of London, Department of Psychology Ethics Committee.)

#### Participants

As Study 2 suggested that the ambiguous sentences task may be sensitive to participants’ interpretation of the task instructions, the current study solicited data only from participants whose first language was English. Eighty-six psychology students (74.4% female), ranging from A-Level (55.8%) through to Postgraduate level (16.3%), completed the study. Participants were aged between 16 and 58 years (*M* = 23.5, *SD* = 9.7), and the majority indicated British or Irish nationality (90.7%).

#### Design

A correlational design was employed. The variables of interest were individual differences in conspiracist ideation, anthropomorphism, and intentional inferences.

#### Materials

*Generic Conspiracist Beliefs scale*. General conspiracist beliefs were again assessed using the GCB. Cronbach’s alpha for the scale was high (*α* = .92).

*Individual Differences in Anthropomorphism Questionnaire*. The IDAQ was again used to measure the degree to which individuals tend to anthropomorphise nonhuman animals and inanimate objects. Internal reliability in the current study was high (*α* = .90).

*Individual differences in intentional inferences*. The 12 ambiguous sentences used in Study 2 to measure individual differences in bias towards intentional inferences were used again in the current study. The general procedure and instructions remained the same. However, minor modifications were made to reflect the web-based interface, and in an effort to avoid the problem of ambiguous responses encountered in Study 2.

An initial web-page presented the 12 sentences in randomised order. Each sentence was accompanied by a small text input field in which participants were asked to type a brief description of the image that came to mind when reading the sentence. On completing this phase of the task, participants clicked a button to move on to a new page, which reiterated the 12 sentences together with the descriptions the participant had entered for each. Participants were instructed, “We’re now going to remind you of the sentences you just read and the answers that you provided. For each of the answers you gave, all we would like you to do is clarify whether the event or action that you imagined was done on purpose or by accident.” To do so, participants selected the appropriate option from a list of options labelled ‘on purpose’ and ‘by accident’. Unlike in Study 2, in the current study a ‘not sure / cannot decide’ option was also provided. This additional response option was included so that participants were not forced to choose one of the former options if they felt unsure of their response, or of the task instructions. ‘Not sure’ responses were excluded from analyses, avoiding the potential introduction of experimenter subjectivity in identifying or coding ambiguous items.

*Demographics*. Participants were asked to indicate their age, nationality, and whether English is their first language (data from non-native-English-speakers were discarded).

#### Procedure

The survey was administered via a computer-based interface. Some participants (approximately one-third of the sample) were tested in person as a group using university computer facilities. The remaining participants were recruited using emailed volunteer requests directed to current A-Level and Postgraduate psychology students, with participants completing the study remotely by accessing the survey online. To avoid priming ideas of conspiracy theories, the word ‘conspiracy’ was not mentioned in the information sheet presented to participants prior to filling in the questionnaire. No time limit was given, though participants were asked to work quickly, answering with their first instincts.

### Results

#### Data screening and descriptives

Raw data are available in S1 Dataset. No cases were missing data for more than two items on the GCB, IDAQ, or intentional inferences measures; thus, none were excluded. No multivariate outliers were identified.

A mean GCB score was calculated for each participant. Scores were approximately normally distributed. On the whole, participants demonstrated slight scepticism towards conspiracist ideas (*M* = 2.71, *SD* = 0.86).

In addition, a mean IDAQ score was calculated for each participant. Scores showed slight positive skew (skewness = 0.42). Participants were generally somewhat disinclined to anthropomorphise, evident from a grand mean slightly below the mid-point of the scale (*M* = 3.54, *SD* = 1.55).

Finally, an intentionality score was calculated for each participant following the procedure used in Study 2. Again, participants generally interpreted significantly more than half (*M* = 7.75, *SD* = 2.41) of the ambiguous sentences as being intentional actions (*t* (85) = 6.70, *p* <. 001, *d* = 0.72). The data were approximately normally distributed about the mean, with scores ranging from 1 to 12.

#### Associations between conspiracist ideation, anthropomorphisation, and intentionality inferences

To first investigate whether individual differences in anthropomorphisation and intentionality bias were related (and thus whether the relationship between anthropomorphisation and conspiracism may be mediated by intentionality biases), the correlation between the two was examined. There was no significant association between intentional inferences and anthropomorphism (*r* (84) = -.10, *p* = .37); participants who indicated higher levels of anthropomorphism were no more likely to interpret the ambiguous sentences as intentional. Given the lack of association between anthropomorphism and the proposed mediator, no further mediation analysis was conducted.

To assess the role of anthropomorphism and intentionality attributions in predicting conspiracist ideation, a multiple regression analysis was conducted with anthropomorphism and intentional inferences as predictors and GCB scores as the criterion. Overall, the model was significant and explained a substantial amount of variance (*F* (2, 83) = 27.72, *p* <. 001, Adj. *R* ^*2*^ = .39). Both predictors contributed significantly to the model (see Table 1). The relationship between intentional inferences and conspiracist ideation was positive and weak, while the relationship between anthropomorphism and conspiracist ideation was positive and more substantial.

**Table 1:** Results of multiple regression with intentional inferences and anthropomorphism predicting conspiracist ideation.

Predictor	*β*	*t*	*p*
Anthropomorphism	.62	7.29	<. 001
Intentional inferences	.19	2.22	<. 05

### Discussion

In line with Study 1, as well as previous research [68,69], the tendency towards endorsing anthropomorphic statements predicted stronger conspiracist ideation. Consistent with the results of Study 2, the number of intentional attributions participants made in response to ambiguous sentences positively predicted their level of agreement with conspiracist statements. Contrary to expectations, however, no association emerged between individual differences in anthropomorphism and biased intentionality inferences, suggesting that anthropomorphism cannot be accounted for by individual differences in the intentionality bias. Rather, the two measures appear to reflect conceptually distinct traits or processes. Neither is the relationship between anthropomorphism and conspiracism mediated by biased intentionality inferences. Multiple regression analysis suggested that both traits predict conspiracist ideation. The relationship between intentionality inferences and conspiracism was small, only verging on statistical significance. However, the successful replication and comparable effect size to that observed in Study 2 suggest that the relationship is reliable. The relationship between anthropomorphism and conspiracism was stronger. The magnitude of the relationship is consistent with previous research [68,69], suggesting that this relationship is also reliable.

## General Discussion

The current research was guided by the speculation that conspiracy theories, which paint events almost exclusively as the product of the conspirators’ intentions, may appear more plausible the more an individual is biased towards seeing intentionality as the primary cause of events in the world in general [31,59,68]. The findings offer support for this idea, as well as shedding light on previous findings concerning trait-anthropomorphism [68,69].

First, the findings indicate a small but reliable association between conspiracist ideation and attributions of intentionality in response to imagined ambiguous actions, such as “She kicked the dog.” Individuals who were more inclined to interpret ambiguous imagined actions as having been intended were more likely to endorse generic conspiracist statements. Conversely, individuals more inclined to interpret ambiguous actions as accidental were more likely to reject conspiracist ideas. While one previous study suggests that people high in conspiracist ideation are more likely to offer an intentional conspiratorial explanation for an ambiguous event [68], the current studies are the first to provide evidence that this may reflect a more general bias towards interpreting *all* ambiguous events—even those with no conspiratorial implications—as having been brought about intentionally.

Second, it was speculated that the previously observed relationship between trait-anthropomorphism and conspiracism [68,69] may be a product of the same bias towards inferences of intentionality. Study 1 replicated the relationship between anthropomorphism and conspiracism, finding a similarly sized correlation as that observed in previous studies. However, Study 3 found no evidence of a relationship between anthropomorphism and biased inferences of intentionality. Scores on both measures significantly predicted conspiracist ideation; no mediation was evident. Thus, the relationship between conspiracism and anthropomorphism cannot be explained as a product of biased inferences of intentionality.

One potential explanation is that anthropomorphism reflects an individual’s broad attitude towards the world and people’s place in it, as opposed to reflecting a low-level bias towards overattributing intentionality. In other words, the intentionality bias measure may capture immediate, intuitive responses to a novel event, whereas anthropomorphism may reflect a more reasoned pre-existing attitude. The two appear to be distinct traits, which independently predict conspiracism.

To speculate further about the relationship between conspiracism and anthropomorphism, previous studies have found conspiracism to correlate with traits reflecting openness to unusual ideas [24,22,76]. In particular, one study reports an association between conspiracism and endorsement of New Age ideas [61]. There is some possible conceptual overlap between measures of New Age beliefs and anthropomorphism. The IDAQ [75], used in the current research, asks “To what extent does the wind have intentions,” for example. Participants may interpret this as reflecting the same sentiment as some New Age beliefs, such as “The whole cosmos is an unbroken living whole…” [61]. Alternatively, anthropomorphism may represent ontological confusions [77]—attributing ontological properties of one category (such as human characteristics) on to another category (such as nonhuman animals or entities)—which have recently been shown to predict conspiracist ideation [78]. In this way, conspiracist ideation may reflect the attribution of human motivations, such as greed and control, on to wider social, political, or physical processes which give rise to conspiracy theories. Future research may examine anthropomorphism in conjunction with a variety of other beliefs or dispositions, such as New Age beliefs and ontological confusion, in order to establish the inter-relationships between these belief systems.

This research was based on the speculation that a domain-general bias towards attributions of intentionality causes conspiracy theories to be evaluated more positively. This seems a reasonable proposal, given evidence that the intentionality bias is a low-level, intrinsic aspect of the cognitive system [31,41,42]. However, the current research is correlational, and as such, does not demonstrate this causal relationship. An alternative possibility is that believing that the world is dominated by conspiracy primes an individual to evaluate any imagined scenario, even outside the context of conspiracy, in terms of intentional agency. The current studies are intended as a preliminary investigation of a novel hypothesis, and the findings appear to provide tentative support. However, it is up to future research to examine the effects in more detail. In particular, research taking an experimental approach is required to establish the direction of causality. Future research may seek to establish whether manipulating the intentionality bias, perhaps via cognitive load [31,40,41], has a direct effect on endorsement of conspiracy theories.

## Supporting Information

S1 Dataset Raw data from Study 1, Study 2, and Study 3. (XLSX)
